# Dentists or Charlatans?

**Published:** 1921-03-12

**Authors:** 


### Dentists or Charlatans?

### THE PUBLIC AND THE NEW BILL.

A great deal has been said, from the professional point of view, as to the probable effect of the Dentists Bill, now awaiting reintroduction into the House of Commons in a slightly modified form. But not very much has been said about the point of view of the public. The problem, as far as the public is concerned, is twofold. On the one hand, something must clearly be done to extend the application of the altogether inadequate dental service throughout the country, especially in regard to the treatment of school children; and, on the other hand, it is almost equally important that the public shall be protected from any undue privileges accorded to unregistered practitioners which may result in the increase of charlatanism.

The British Dental Association, which claims to be the only body representing the profession as a whole, has now issued a statement which seems to put the matter very fairly and straightforwardly. The next difficult problem, it points out, is the consideration to be given to the existing unregistered practitioners, the dilemma being that, while many by long practice had acquired vested interests, the charlatanism of others constituted the chief reason for the Bill, and ought to be suppressed, rather than given legal sanction by recognition.

The Bill attempts to solve this problem in a rough-and-ready way by placing on the Dentists' Register all those who have been practising for five years, and prescribing an examination test before admission for those who have been practising for a less period. It says much for the public spirit of the majority of registered dentists that they should have been induced to surrender as much as they have without bickering and recrimination. Nevertheless, they do feel that it is in the public interest that a distinction should be drawn between those who have passed some sort of minimum test and those who have no credentials whatsoever.

The claims of vested interest could be sufficiently met by the institution of a "list of exempted persons," any of whom, by passing a practical test to show that they might be regarded as a benefit instead of a danger to the public, could then be placed on the register itself. It is to be hoped that an amendment on these lines will be accepted by the Government or by Parliament. The assertion has been made that the Bill is the forerunner of an expensive public dental service; such a statement is a distortion of the facts. The national health needs good dentistry; the public needs a sufficient supply of trained dentists; large sections of the public need protection against quackery. This Bill is intended to make provision for those needs, and comes as near doing so as any Bill is ever likely to do.

